# Co action of CFTR and AQP1 increases permeability of peritoneal epithelial cells on estrogen-induced ovarian hyper stimulation syndrome

**DOI:** 10.1186/1471-2121-13-23

**Published:** 2012-08-28

**Authors:** Pei-Yin Jin, Yong-Chao Lu, Ling Li, Qin-Fu Han

**Affiliations:** 1 Department of Cardiovascular Medicine, The First Affiliated Hospital of Zhengzhou University, Zhengzhou, Henan, 450052, China; 2 Department of Reproductive Technology, Henan Provincial institute for Polulation and Family Planning Research, Zhengzhou, Henan, 450002, China; 3 Department of Cardiovascular Medicine, The People’s Hospital of Anyang, Anyang, Henan, 455000, China

**Keywords:** CFTR, AQP1, Estrogen, Ovarian hyper stimulation syndrome

## Abstract

**Background:**

Ovarian hyper stimulation syndrome (OHSS) is an iatrogenic complication associated with fertility drugs. It is characterized by increased vascular permeability and substantial fluid shift with accumulation in the body cavity. The pathogenesis of OHSS remains obscure, and no definitive treatments are currently available.

**Results:**

Using western blot and short-circuit current (Isc) techniques, we investigate the potential coactions of analysis in cystic fibrosis transmembrane conductance regulator (CFTR) and aquaporin 1 (AQP1) on the hyper permeability of body cavity peritoneal epithelial cells in the pathogenesis of OHSS. The rats develop OHSS symptoms, with the up regulation of both CFTR and AQP1 expression and enhanced CFTR channel activity in peritoneal epithelial cells, can also be mimicked by administration of estrogen, alone in ovariectomized rats. Administration of progesterone suppresses CFTR activity, OHSS symptoms as well as CFTR and AQP1 expression. Besides, AQP1 inhibitor, HgCl_2_, can suppress CFTR channel activity. Therefore, antisera against CFTR or AQP1 to OHSS animals may result in alleviation of the symptom.

**Conclusion:**

This study confirms the coactions of CFTR and AQP1 play a critical role in the development and progression of increased peritoneal epithelial permeability in severe OHSS. These findings may provide grounds for ameliorating assisted reproduction treatment strategy to reduce the risk of OHSS in *in vitro* fertilization (IVF).

## Background

Ovarian hyper stimulation syndrome (OHSS) is a severe complication of ovulation induction in *in vitro* fertilization (IVF) treatment. The incidence of OHSS occurs in about 0.1–4% of ovulation induction treatments [1], is increasing around the world through the expansion of infertility treatments. OHSS is characterized by massive cystic enlargement of the ovaries associated with third space fluid shift. This can result in the formation of ascites and pleural effusion in severe conditions due to increased peritoneal epithelial permeability [2]. Severe OHSS can be accompanied by hypercoagulability, thromboembolic phenomena, adult respiratory distress syndrome, and even death. Although this syndrome has been identified for a long time, the exact mechanism of OHSS induction is not clear. An increased epithelial permeability is the initial change leading to OHSS [3]. Many cytokines and vasoactive and angiogenic factors, such as vascular endothelial growth factor (VEGF), have been implicated as major mediators of the pathogenesis of capillary leakage and endothelial damage in OHSS [4-7].

Fluid effluxing across epithelial cells is followed by ion movements. Ion movements play a crucial role in the movement of water across epithelial cells, as water fluctuation depends on the opening and closing of aquaporins (AQPs) actively and inactively transported as it follows ion movements according to osmotic gradients [8]. Rapid passage of fluid into luminal spaces, as seen in OHSS, may be a consequence of abnormal ion transport across the epithelial cells. Several pathological conditions, such as cholera-induced diarrhea, in which there is massive fluid efflux of ion and water across epithelial membranes, are mediated by altered expression and function of transepithelial ion channels, particularly cystic fibrosis transmembrane conductance regulator (CFTR) [9]. Cystic fibrosis is manifested by mutations in CFTR [10], the most common lethal genetic disease in Caucasians, with a hallmark defect in epithelial electrolyte and fluid transport throughout the body [11]. A previous study only focused on the reproductive system effect of CFTR on OHSS, but did not investigate CFTR in the peritoneal epithelial cells [12]. Meanwhile, in the process of fluid effusion, water efflux involves a water (AQP1) pathway by which ion and other osmotic gradients drive water movement [8]. So the coactions of ion channels and AQP1 such as CFTR [12] are important in the process of fluid effusion.

CFTR is expressed in most epithelial cells including the airways and gastrointestinal and reproductive tracts [13,14]. Its expression is known to be regulated by ovarian hormones [15,16], upregulated by estrogen, and down regulated by progesterone [17]. Cyclic changes in CFTR expression and its channel activity have been correlated with cyclic changes in uterine fluid volume [18,19]. Meanwhile, the water channel AQP1 is expressed strongly throughout microvascular endothelia except the central nervous system, such as in the kidneys, lungs, skins, secretory glands, skeletal muscles, pleura and peritoneum [20,21]. AQP1 is involved in fluid transport [20,21], and facilitates water movement from capillaries into the peritoneal cavity [22]. Its expression is also regulated by ovarian hormones [23,24]. It has also been well established that estrogen levels are highly elevated during ovarian hyper stimulation, with excessively high levels observed in patients with OHSS [25,26]. We, therefore, hypothesized that increased estrogen levels during ovarian hyper stimulation may lead to upregulated CFTR and AQP1 expression and channel activity, resulting in the elevated epithelial secretory activity. The most prominent feature of OHSS is marked with ascites or even pleural effusion almost always associated with the administration of human chorionic gonadotropin (hCG) [2,27]. So we chose peritoneal epithelial cell model for the study of coactions of CFTR and AQP1 in OHSS development.

## Results

### Up regulation of CFTR and AQP1 expression in peritoneal epithelial cells

**Figure 1:**
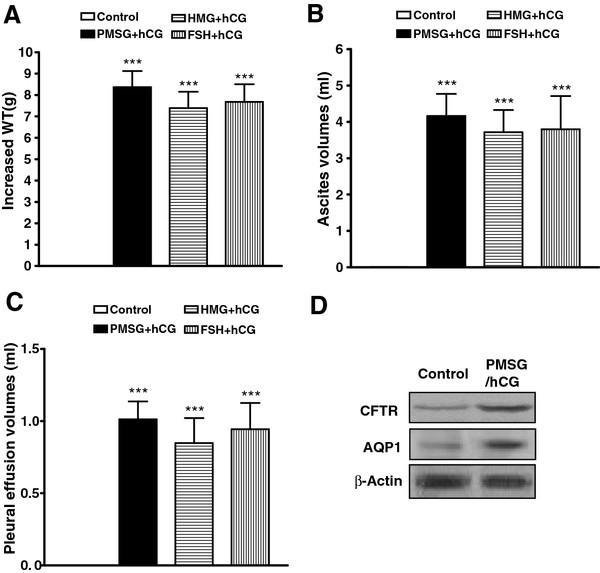
**Induction of OHSS rat model and upregulation of CFTR and AQP1 expression in peritoneal epithelial cells.** Hyperstimulated rats given different combinations of gonadotropins showed significantly enhanced WT increase, ascites volumes and pleural effusion volumes compared with the controls (**A**, **B** and **C**; n = 5) with corresponding enhancement of CFTR and AQP1 protein expression by western blot in PMSG- and hCG-induced OHSS rat peritoneal epithelial cells. The Pixel Value for CFTR: Control 265488, PMSG/hCG 765729; The Pixel Value for AQP1: Control 150834, PMSG/hCG 348630; The Pixel Value for β-Actin: Control 980058, PMSG/hCG 971052 (**D**). ***P< 0.001 (compared with the control).

Combinations of gonadotropins including HMG, FSH, or PMSG in conjunction with hCG were used to trigger OHSS. In our study, rat hyper stimulation was defined by the observation of either fluid accumulation in the peritoneal cavity and thorax, or increased body weight (WT) in contrast to the saline-treated control rats (Figure 1 A-C). Body weight was measured, and ascites was collected by performing a lower midline incision and completely draining the intra-abdominal fluid into a flask. After ascites was collected, the diaphragm was cut off by scissors and pleural effusion volume was also collected. The effusion volumes were then measured with a 1-ml tuberculin syringe respectively. Most prominently, the increase of ascites volume and pleural effusion volume in the hyper stimulated rats was significantly different from that in the controls (Figure 1 B and C). This was associated with elevated CFTR and AQP1 expression in peritoneal epithelial cells (Figure 1 D).

### Estrogen upregulated CFTR and AQP1 expression and induced OHSS in rats

**Figure 2:**
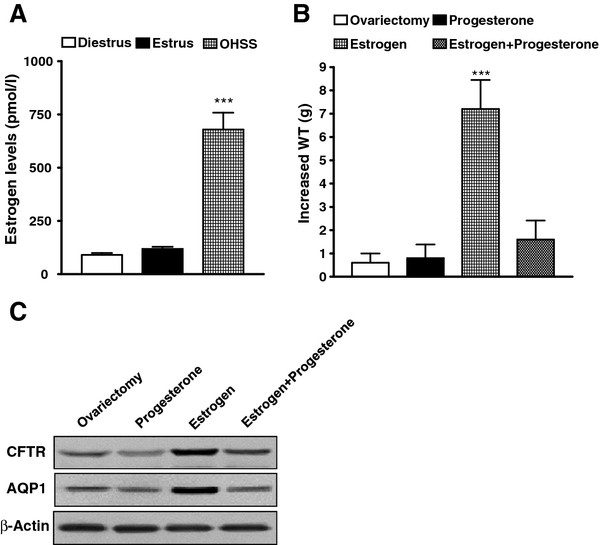
**Estrogen upregulates CFTR and AQP1 expression in peritoneal epithelial cells and induces OHSS in rats.** Serum estrogen concentrations in OHSS were significantly different from control rats at both estrous and diestrous stages of the estrous cycle (**A**). ***P< 0.001 (n = 6, 6, and 6 for diestrus, estrus, and OHSS, respectively). Comparison of increased WT of rat 30 d after ovariectomy (control) and after treatment with progesterone or estrogen for 5 d (n = 5/group), showing significantly increased WT by estrogen (**B**). ***P< 0.001 (compared with the control). Western blot results showed downregulation of CFTR and AQP1 expression by progesterone and upregulation expression by estrogen in peritoneal epithelial cells of ovariectomized rats. The Pixel Value for CFTR: ovariectomy 260546, Progesterone 202730, Estrogen 885290, Estrogen+Progesterone 363422; The Pixel Value for AQP1: ovariectomy 186484, Progesterone 101092, Estrogen 877020, Estrogen+Progesterone 160054; The Pixel Value for β-Actin: ovariectomy 708508, Progesterone 707448, Estrogen 707982, Estrogen+Progesterone 709654 (**C**).

Estrogen levels were measured in our PMSG/hCG-induced OHSS rats and those control rats at both estrous and diestrous stages of the estrous cycle. More than 5-fold increase in estrogen levels was found in the OHSS rats compared with that in the control (Figure 2 A). We also found that hyper stimulation did not produce OHSS symptom in ovariectomized rats. However, treatment of ovariectomized rats with estrogen (10 μg/kg·d), but not progesterone (25 mg/d) for 5 d, induced WT increase compared with ovariectomized control (*P* < 0.0001; Figure 2 B) and increased CFTR and AQP1 expression in peritoneal epithelial cells (Figure 2 C) similar to the OHSS rat model. Furthermore, progesterone suppressed the action of estrogen on OHSS induction and CFTR and AQP1 expression up regulation (Figure 2 B and C). These results suggest that the ovarian sex hormone, estrogen, but not progesterone, is responsible for the up regulation of CFTR and AQP1 leading to OHSS.

### Effect of OHSS on peritoneal epithelial CFTR channel activity in rats

**Figure 3:**
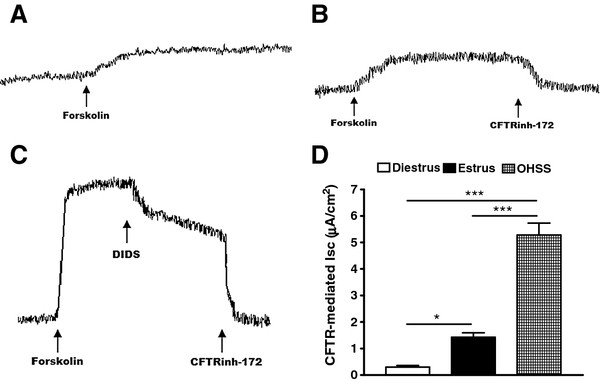
**Effect of OHSS on peritoneal epithelial CFTR channel activity.** CFTR-mediated peritoneum anion secretion measured by Isc in the presence of an epithelial Na^+^ channel blocker, amiloride (10 μM), showed an elevated forskolin (10 μM) response in PMSG-/hCG-induced OHSS peritoneal epithelial cells (**C**) compared with the controls at diestrous (**A**) and estrous (**B**) stages of the sexual cycle (**D**). *P< 0.05, ***P< 0.001 (n = 4/group, compared with the diestrus).

We compared CFTR activity between freshly isolated peritoneal epithelial cells of normal and OHSS rats by Isc measurement. In the presence of an epithelial sodium channel blocker, amiloride (to exclude a possible contribution of Na^+^ absorption to the current observed), the forskolin-induced Isc from the peritoneal epithelia of OHSS rats (PMSG plus hCG) was substantially increased compared with that in untreated controls at the diestrous or estrous stages of the sexual cycle (Figure 3). The cAMP-dependent Isc could be blocked by a CFTR specific inhibitor-172 (Calbiochem, La Jolla, CA USA; Figure 3 A-D), confirming that the observed cAMP-activated Isc reflects CFTR channel activity. The results showed that CFTR channel activity was enhanced in OHSS.

### Effect of estrogen and progesterone on the peritoneal epithelial CFTR channel activity

**Figure 4:**
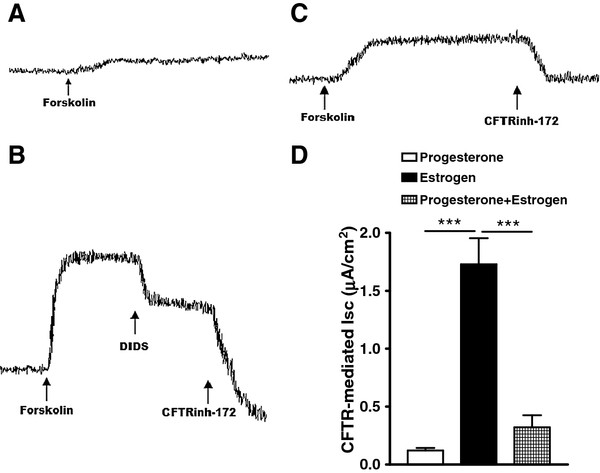
**Effect of estrogen and progesterone on the peritoneal epithelial CFTR channel activity.** Progesterone treatment (25 mg/d·rat) of female rats for 5 d did not induce CFTR-mediated Isc in the peritoneal epithelial cells (**A**), but estrogen treatment (10 μg/kg body weight·d) did (**B**). Besides, progesterone treatment suppressed estrogen induced CFTR-mediated Isc (**C**). Statistical analyses of forskolin-activated CFTR-mediated Isc response showed significant CFTR activity in the peritoneum of estrogen-treated rats (**C**). ***P< 0.001 (n = 12).

The effect of exogenously administrated estrogen and progesterone for 5 d on peritoneum CFTR channel activity was examined. Isc measurements showed that forskolin-induced or CFTR-mediated Isc in estrogen-treated peritoneum (Figure 4 B and D) was significantly higher than that in progesterone-treated ones (Figure 4 A and D). Besides, progesterone suppressed estrogen induced CFTR-mediated Isc in estrogen-treated peritoneum (Figure 4 C and D).

### Progesterone downregulated CFTR and AQP1 expression and suppressed OHSS rats

**Figure 5:**
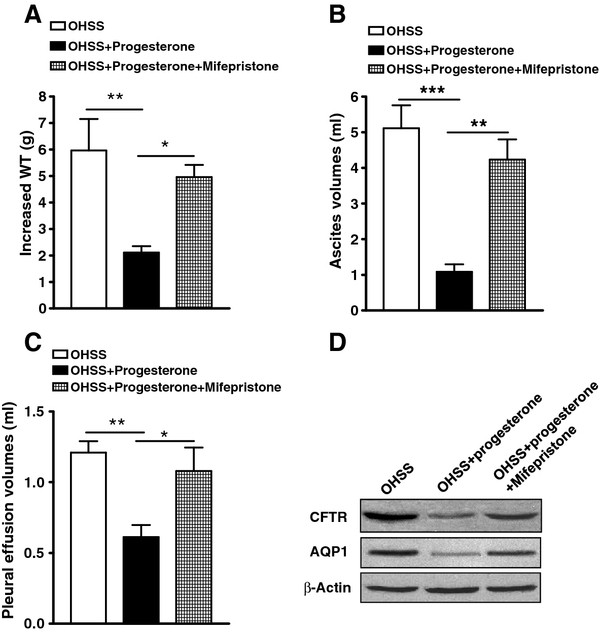
**Effect of progesterone on the expression of CFTR and AQP1 expression in peritoneal epithelial cells and OHSS symptoms in rats.** Statistical comparison of WT increase (**A**), ascites volume (**B**), and pleural effusion volume (**C**) in PMSG-/hCG-induced OHSS rats and OHSS rats [treated with progesterone, 25 mg/d·rat; or treated with both progesterone (25 mg/d·rat) and mifepristone (diluted in corn oil, 10mg/d·rat, irrigation daily), n = 5/group]. *P < 0.05, **P < 0.01, *** *P <* 0.001. Western blot results showed that expression of CFTR and AQP1 proteins in peritoneal epithelial cells were suppressed by progesterone in the OHSS rats. The Pixel Value for CFTR: OHSS 1005806, OHSS+progesterone 223260, OHSS+progesterone+mifepristone 394564; The Pixel Value for AQP1: OHSS 424325, OHSS+progesterone 183016, OHSS+progesterone+mifepristone 248076; The Pixel Value for β-Actin: OHSS 821654, OHSS+progesterone 824855, OHSS+progesterone+mifepristone 823090 (**D**).

Progesterone treatment (25 mg/rat·d) of PMSG/hCG-induced OHSS rats significantly reduced WT increase, ascites volume and pleural effusion volume (Figure 5 A-C). Progesterone treatment of OHSS rats also significantly reduced CFTR and AQP1 protein expression (Figure 5 D). Besides, a selective progesterone antagonist, mifepristone irrigation significantly reduced the reverse of progesterone to OHSS symptoms of rats as well as CFTR and AQP1 expression reduction in peritoneal epithelial cells (Figure 5 A-D). It may be the reason why progesterone was found to significantly lower the incidence of OHSS in IVF cycles in a few clinical situations for luteal support during assisted reproductive technology (ART) [28-30].

### Coaction of the peritoneal epithelial CFTR and AQP1 channel activity in the development of OHSS

**Figure 6:**
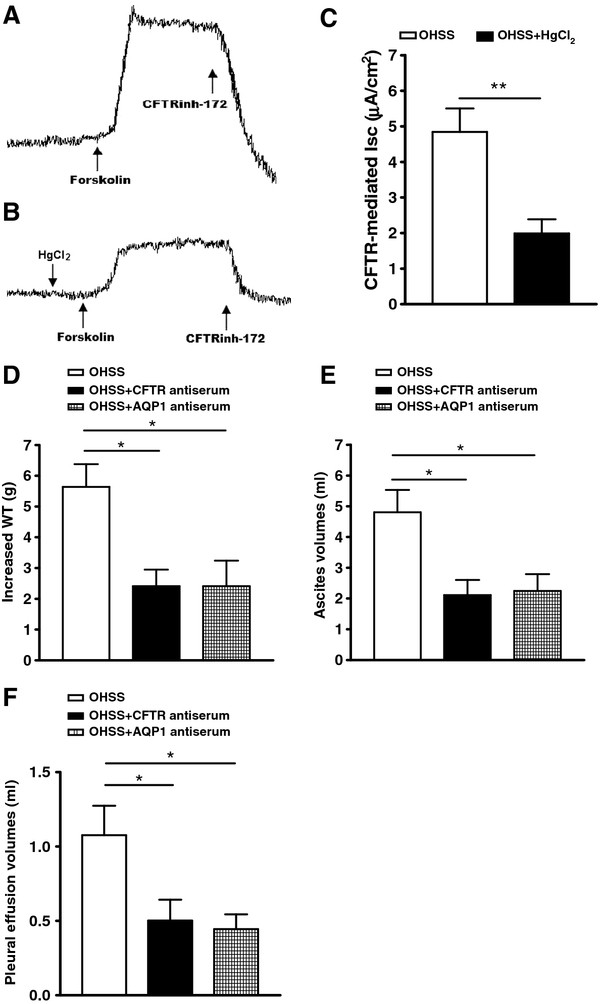
**Coactions of CFTR and AQP1 in the excessive fluid transport in OHSS.** The HgCl_2_ pre-treatment (30μ M) suppressed CFTR-mediated Isc in the peritoneal epithelial cells (**A**, **B** and **C**), showing Coaction of CFTR and AQP1 activity in the peritoneal epithelial cells. **P< 0.01 (n = 6). Noncycling PMSG-/hCG-induced OHSS rats treated with 300 μl CFTR antiserum or 400μl AQP1 antiserum (1:100 dilution, vol/vol, in buffer) significantly reduced WT increase (**D**), ascites volumes (**E**) and pleural volumes (**F**) compared with the heat-inactivated normal rat serum (NRS)-treated controls. *P < 0.05 (n = 6).

We found that AQP1 inhibitor (HgCl_2_) could suppress the CFTR activity in the freshly isolated peritoneal epithelial cells of OHSS rats by the short circuit current (Isc) measurement. For the OHSS rats (PMSG plus hCG), in the presence of an AQP1 inhibitor, HgCl_2_ (to block water exocrine), the forskolin-induced Isc from the peritoneal epithelial cells of OHSS rats (PMSG plus hCG) was substantially decreased compared with that in untreated controls of the OHSS rats (Figure 6 A-C). Furthermore, treatment of noncycling PMSG-/hCG-induced OHSS rats (to exclude any cyclic influence) with 300 μl CFTR antiserum (1:100 dilution, vol/vol, in buffer; Zymed Laboratories) or 400μl AQP1 antiserum (1:100 dilution, vol/vol, in buffer; Santa Cruz Biotechnology) by IP injection to suppress the function of CFTR or AQP1 resulted in the elimination of body WT increase, ascites volumes and pleural effusion volumes (Figure 6 D-F).

## Discussion

In addition to being a chloride channel, CFTR, may function as a regulator of other channels, including aquaporin water channels (channels that allow rapid flow of water across epithelial membranes) and epithelial sodium channels. Enhancement of aquaporin channel activity [31] and suppression of epithelial sodium channel-mediated absorptive activity [32] that results in net increases in electrolyte and fluid secretion may also contribute to the pathogenesis of OHSS.

To test this hypothesis, we developed the OHSS rat model by using several combinations of gonadotropins as previously described [33]. There was a marked increase in fluid accumulation in the peritoneal cavity and pleural cavity (Figure 1 B and C). Therefore, the increased body WT in the hyperstimulated rats was significantly different from those in the controls (Figure 1 A). Enhanced CFTR and AQP1 expression in body cavity peritoneal epithelial cells was observed in the OHSS rats (Figure 1 D). Thus, up-regulated CFTR and AQP1 expression in peritoneal epithelial cells is correlated with the fluid accumulation observed in the body cavity of OHSS animals. It has been well documented that OHSS patients have high plasma estrogen levels [26]. It is possible that hyper stimulation of ovaries leads to high production and release of estrogen, which is responsible for the upregulation of CFTR and AQP1 observed. This may explain why OHSS may not occur or may become severe despite high estrogen levels in some individuals who could have CFTR mutations and defective CFTR function.

Estrogen levels had been monitored in the induced OHSS rats as well as in the controls at both estrous and diestrous stages of the estrous cycle. The results showed that estrogen levels increased over 6-fold in the OHSS rats compared with that in the controls (Figure 2 A), which is consistent with other OHSS animal models [33]. The relationship between elevation of estrogen alone and OHSS had been investigated by developing of OHSS symptoms in ovariectomized rats. It confirmed that hyper stimulation did not produce OHSS symptoms in these ovariectomized rats unless treated with estrogen, but not progesterone, for 5 days (Figure 2 B). Furthermore, progesterone suppressed estrogen induced OHSS symptom as well as CFTR and AQP1 expression upregulation (Figure 2 B and C). Exogenous estrogen induced WT increase as well as CFTR and AQP1 expression upregulation in ovariectomized rats, similar to those in the OHSS rat model without ovariectomy (Figure 2 B and C). It should be noted that although estrogen drastically increased CFTR and AQP1 expression, progesterone completely suppressed both of them in ovariectomized rats. This is consistent with previous results obtained in ovary-intact animals showing upregulation and downregulation of CFTR by estrogen and progesterone, respectively [17]. OHSS symptoms and CFTR upregulation in peritoneal epithelial cells were also observed in ovary-intact rats treated with high levels of estrogen, but not progesterone (Figure 2 B and C). The serum level of exogenously administrated estrogen was similar to the estrogen levels in OHSS rats (Figure 2 B and C). These results suggest that the ovarian sex hormone, estrogen, but not progesterone, is responsible for the upregulation of CFTR and AQP1 leading to OHSS. These results support the clinical observation that OHSS rarely develops in individuals undergoing ovarian hyper stimulation in the absence of high estrogen levels [26].

To confirm that up regulation of CFTR indeed leads to enhancement of its channel activity, from where excessive epithelial fluid secretion derives, we compared functional CFTR activity between freshly isolated peritoneal epithelial cells of normal and OHSS rats by the Isc measurement [34]. To exclude a possible contribution of Na^+^ absorption to the current, we used amiloride, an epithelial sodium channel blocker. The results revealed that the forskolin-induced Isc from the peritoneal epithelial cells of OHSS rats was substantially increased compared with that of untreated controls at the diestrous or estrous stage of the sexual cycle (CFTR is known to be minimally and maximally expressed, respectively). The cAMP-dependent Isc could be blocked by a specific CFTR inhibitor-172 (Figure 3 A-D), which has been demonstrated to inhibit CFTR-mediated, cholera toxin-induced fluid secretion [35]. It confirmed that the observed cAMP-activated Isc reflects CFTR channel activity. The effect of exogenously administrated estrogen and progesterone for 5 d on peritoneal CFTR channel activity was also examined. Isc measurements showed that forskolin-induced or CFTR-mediated Isc in estrogen-treated peritoneum was significantly higher than that of progesterone-treated (Figure 4 A and B). Progesterone suppressed estrogen induced CFTR-mediated Isc in peritoneum (Figure 4 C). Taken together, it appears that up regulation of CFTR and AQP1 due to elevated estrogen may be responsible for the excessive fluid transport and accumulation in OHSS. Therefore, it may be possible to alleviate OHSS symptoms through suppressing CFTR or AQP1 expression or through suppressing CFTR or AQP1 function. To validate this hypothesis, we first used progesterone, as it was shown to suppress CFTR expression in previous studies [17] and in the present study as well. Indeed, progesterone treatment of OHSS rats also eliminated peritoneal and pleural fluid accumulation and significantly reduced CFTR and AQP1 protein expression (Figure 5 A-D). The progesterone selective progesterone antagonist, mifepristone, was found to suppress the action of progesterone (Figure 5 A-D). Interestingly, progesterone has been used in a few clinical situations for luteal support during ART, in which it was found to significantly lower the incidence of OHSS in IVF cycles. However no explanation for such an effect was published [28-30]. In summary, progesterone may suppress the expression of CFTR and AQP1 to alleviate the symptom of OHSS.

To confirm the coactions of CFTR and AQP1 in the pathogenesis of OHSS, the cAMP-dependent Isc was used to measure CFTR channel activity affected by AQP1. The results showed that the cAMP-dependent Isc could also be reduced by an AQP1 inhibitor, HgCl_2_ (Figure 6 A-C), which has been demonstrated to inhibit AQP1-mediated water transport [36]. This confirmed that AQP1 can affect CFTR channel activity. Furthermore, treatment of PMSG-/hCG-induced OHSS rats injected with 300 μl CFTR antiserum resulted in the elimination of the body WT increase about 3.23 g, the peritoneal fluid accumulation about 2.69 ml and the pleural fluid accumulation about 0.58 ml. Injection of 300 μl AQP1 antiserum resulted in the elimination of the body WT increase about 3.23 g, the peritoneal fluid accumulation about 2.56 ml and the pleural fluid accumulation about 0.64 ml (Figure 6 D-F). The ability to alleviate OHSS symptoms through interfering with CFTR or AQP1 function confirmed a critical role of the coactions of CFTR and AQP1 in OHSS pathogenesis. It also suggested that these treatments may be a preventive measure for OHSS during ART.

In this study, we demonstrated that up regulation of both CFTR and AQP1 by either excessive estrogen or ovarian hyper stimulation leads to the development of OHSS symptoms in rodents. Considering the observed high levels of estrogen, exceeding normal physiological levels, in patients undergoing ovarian hyper stimulation [25,26] and regulation of CFTR and AQP1 expression by ovarian hormones in humans [14], the present findings in OHSS rodent models are of strong clinical relevance, suggesting the coactions of CFTR and AQP1 in the pathogenesis of OHSS. OHSS appears to be the result of abnormally up regulating CFTR as well as AQP1. This leads to excessive fluid shift and accumulation in the peritoneal cavity and pleural cavity, which can be life threatening. It is because that fluid transport throughout the body due to water effusion following ion transportation.

## Conclusions

Taken together, this study confirms the coactions of CFTR and AQP1 in regulating body electrolyte and fluid secretion. An abnormality in either expression or function, could result in lethal conditions, as seen in OHSS.

## Methods

### Animals

Prior approval of the experimental protocols was obtained from the local ethics committee at Zhengzhou University for use of animals in experiments. The Sprague–Dawley (S-D) rats were used in this study. Animals were maintained in the Laboratory Animal Service Center of Zhengzhou University before experiments and were kept in a temperature-controlled room with a 12L:12D cycle and with food and water *ad libitum*. All animal experiments were conducted in accordance with the university Laboratory Animals Service Center’s guidelines on animal experimentation.

### Ovariectomy

The animals were 13 weeks of age (when rats reach sexual maturation), weighing about 250–300 g. General anesthesia was administered using intraperitoneal (IP) injection of a mixture of 2% xylazine hydrochloride (10 mg/kg) (Alfasan, Woerden, Holland) and ketamine hydrochloride (80 mg/kg) (Alfasan). With the use of sharp dissecting scissors, both oviducts were exposed, one at a time, after careful dissection and bilateral oviductal ligation were performed, and ovaries were removed. The uterine horns were gently put back into the peritoneal cavity. The muscle layers were approximated with absorbable suture, skin incisions were closed using surgical clips, and adequate postoperative care was given. Rats were kept for at least 30 d to recover and adjust before additional experiments.

### OHSS induction

Female S-D rats at 13 weeks old were divided into four groups (n=5/group). All rats were weighed before the experiment. To prepare the OHSS model, the rats are stimulated with human menopausal gonadotropin (HMG, Serono Laboratories, Aubonne, Switzerland), follicle stimulating hormone (FSH, Organon NV, Oss, The Netherlands), or pregnant mares’ serum gonadotrophin (PMSG, Sigma-Aldrich Corp., St. Louis, MO, USA) in conjunction with hCG (Organon NV) as previously reported [33]. Gonadotropins were administered by IP injections. PMSG (300 IU/kg body weight), HMG (75 IU/rat·d), and FSH (75 IU/rat·d) were injected for 4 d consecutively; on the fifth day, rats received 300 IU hCG/rat. Control animals received phosphate buffered saline (PBS) for the same period. Serum concentrations of estradiol is also increased.

### Western blot analysis

Tissue samples, peritoenal biopsy, were lysed with modified RIPA buffer (50 mM Tris–HCl, pH 7.4; 150 mM NaCl, 1% NP40, 0.25% Na-deoxycholate, 1 mM EDTA) supplemented with 1mM PMSF to inhibit proteolytic enzyme. The whole cell lysates containing equal amounts of protein (20-30μg) were separated on 10-12% sodium dodecyl sulphate polyacrylamide gel electrophoresis (SDS-PAGE) and transferred to Hybond-ECL nitrocellulose membranes (Amersham, USA). Membrane was blocked using 4% skimmed milk and then incubated with anti-CFTR antibody (Zymed Laboratories, San Fransisco, CA, USA), β-Actin (Santa Cruz Biotechnology, Santa Cruz, CA, USA) and anti-AQP1 antibody (Santa Cruz Biotechnology). The blots were incubated with HRP-conjugated goat anti-rabbit secondary antibodies (Santa Cruz Biotechnology) and were visualized by Enhanced Chemiluminescence (ECL) (Amersham Biosciences, USA) following light sensitive films (Fuji, Japan) development. Integrated density values were calculated using Alpha Imager 3400 (Alpha InnoTech, USA). The actual values of the western blot bands were quantified using MetaMorph image analysis software (Universal Imaging Corporation, USA). And the quantities of the protein were valued by pixel value. All experiments were repeated at least three times and the representative results were presented.

### Short-circuit current (Isc) measurement

Isc measurement has previously been described [34]. In brief, freshly removed parietal peritoneum from which the muscular layers had been removed were clamped vertically between two halves of the Ussing chamber. The peritoneal epithelial cells were bathed on both sides with Krebs-Henseleit solution that was maintained at 37 C. The Krebs-Henseleit solution had the following composition: 117 mM NaCl, 4.7 mM KCl, 2.5 mM CaCl2, 1.2 mM MgCl2, 24.8 mM NaHCO3, 1.2 mM KH2PO4, and 11.1 mM glucose and it was bubbled with 95% O2 and 5% CO2 to maintain the pH at 7.4. Drugs were added directly to the apical or basolateral side of the epithelial cells. The transepithelial potential differences exhibited by the epithelial cells were measured by the Ag/AgCl electrodes (World Precision Instruments, Sarasota, FL, USA) connected to a preamplifier, which was connected to a voltage clamp amplifier (DVC 1000, World Precision Instruments). The change in Isc was defined as the maximal rise in Isc after agonist stimulation and was normalized as current change per unit area of the peritoneal epithelial cells (microamperes per square centimeter).

### Hormonal measurement

Serum estrogen concentrations were measured using an electro-chemiluminescence immunoassay by means of the Elecsys Estradiol 11 kit with the automated Elecsys Immunoanalyzer (Roche, Mannheim, Germany).

### Statistical analysis

Data are presented as the means ± SEM. Multiple comparisons and differences between groups were analyzed using the one-way ANOVA test, and paired data were analyzed by unpaired *t* test. *P* < 0.05 (two-tailed) was considered statistically significant. Analyses were carried out with PRISM (GraphPad, Inc., San Diego, CA, USA).

## Competing interests

The authors declare that they have no competing interests related to the manuscript.

## Authors’ contributions

L.L. conceived and designed the experiments, and revision of the paper critically for important intellectual content and final approval of the version to be published. P.Y.J. performed the experiments. Y.C.L and P.Y.J revised the paper. P.Y.J. and Q.F.H. analyzed the data. P.Y.J., Q.F.H. and L.L. drafted the article. All authors read and approved the final manuscript.
